# Social participation in older women and men: differences in community activities and barriers according to region and population size in Canada

**DOI:** 10.1186/s12889-019-7462-1

**Published:** 2019-08-16

**Authors:** Daniel Naud, Mélissa Généreux, Jean-François Bruneau, Aline Alauzet, Mélanie Levasseur

**Affiliations:** 1 Research Centre on Aging, Centre Intégré Universitaire de Santé et de Services Sociaux de l’Estrie — Centre Hospitalier Universitaire de Sherbrooke (CIUSSS de l’Estrie – CHUS), 1036 Belvedere South, Sherbrooke, Quebec J1H 4C4 Canada; 2 Department of Community Health Sciences, Université de Sherbrooke, Pavillon Gérald Lasalle, 3001 12th Avenue North, Sherbrooke, Quebec J1H 5N4 Canada; 3 Department of Applied Geomatics, Université de Sherbrooke, 2500 University Blvd, Sherbrooke, Quebec J1K 2R1 Canada; 4 Institut français des sciences et technologies des transports, de l’aménagement et des réseaux / Département Transport, Santé, Sécurité / Laboratoire Ergonomie et Sciences Cognitives pour les Transports, Site de Lyon-Bron, Cité des mobilités, 25 avenue François Mitterrand, Case 24, 69675 BRON cedex, France; 5 School of Rehabilitation, Faculty of Medicine and Health Sciences, Université de Sherbrooke, 3001 12th Avenue North, Sherbrooke, Quebec J1H 5N4 Canada

**Keywords:** Community integration, Environment and public health, Older adults, Province, Population spatial distribution, Canada, Social environment, Material environment, Local neighborhood

## Abstract

**Background:**

Social participation is a modifiable health determinant influenced by physical and social aspects of the environment. Little is known about aging women’s and men’s community activities and barriers according to region and population size. This study compared social participation, desire to participate more, and perceived barriers of aging women and men by Canadian region and population size.

**Methods:**

A secondary analysis of the 2008–2009 cross-sectional Canadian Community Health Survey - Healthy Aging was done with 16,274 respondents aged 65+. Respondents were grouped into five regions [Atlantic, Quebec, Ontario, Prairies and British Columbia] and five population size groups [rural (< 1000 inhabitants); small urban (1000-29,999); medium urban (30,000-99,999); large urban (100,000-499,999); and metropolitan (≥500,000) areas]. Social participation was estimated by monthly frequencies of engagement in community activities. If they desired to participate more, respondents were asked to identify barriers to their participation from a list of 13 reasons.

**Results:**

There were no differences in total social participation between regions but Prairies and Quebec respondents had the highest and lowest frequency, respectively, of activities with family and friends (5.4 and 4.3 activities/month; *p* = 0.01). Medium urban centers had the highest participation and metropolises, the lowest (17.4 vs 14.3 activities/month; *p* < 0.01). About one fourth of all respondents wanted to participate more, regardless of region or population size. Overall, women wanted to participate more than men (26.6 vs 20.7%; *p* < 0.001), especially in Ontario (28.3 vs 21.1%; *p* < 0.001) and British Columbia (30.1 vs 22.9%; *p* < 0.001). Men in Quebec were less likely than men in other regions to report “personal responsibilities” as a barrier to participation (*p* < 0.001). Men were more likely than women to report being “too busy”, especially in rural areas (27.1 vs 6.5%; *p* < 0.001). Rural women were more likely than rural men to be constrained by transportation problems (15.1 vs 1.2%, *p* < 0.001). Unavailability of activities was more of a constraint in rural areas than metropolises (13.6 vs 6.0%, *p* < 0.001).

**Conclusions:**

Overall, there were no practical differences between women’s and men’s social participation. However, unavailability of activities and transportation problems suggest that local initiatives and further research on environmental characteristics are required to foster aging Canadians’ participation.

**Electronic supplementary material:**

The online version of this article (10.1186/s12889-019-7462-1) contains supplementary material, which is available to authorized users.

## Background

The reshaping of the world’s demography requires a better understanding of community environments and their influences on health. Due to increased longevity, decreased fertility and the aging of baby boom generations [1], older adults make up a growing portion of the population. In Canada, older adults are not evenly distributed across provinces. In 2014, the population aged 65 and over was estimated to be as high as 18.3% of the total population in Nova Scotia and New Brunswick and as low as 11.4% in Alberta [2]. The population aged 65 and over is expected to reach about 23% in Canada as a whole by 2038 and exceed one third of the population in the Atlantic provinces [3]. These distributions raise concerns not only about income security and the labor force but also about the provision of health care [4]. To decrease the burden on health care systems, it is important to develop and promote innovative solutions for improving the health and well-being of aging populations.

Social participation is defined as a person’s involvement in social activities that provide interactions with others in the community [5]. It is an important dimension of active aging and a determinant of health [6]. A recent meta-analysis demonstrated that people with strong social relationships have a 50% increased likelihood of survival [7]. Social participation is also positively associated with decreased mortality [8], disability [9], depression [10] and cognitive decline [11], and shorter hospital stays [12]. As the social participation of older adults is modifiable, actions can be taken to increase it.

Social participation is influenced by physical and social aspects of the environment. Physical aspects include urban design, traffic density and speed, esthetics and crime [13]. Greater proximity to resources is also associated with greater social participation of older adults [14]. While the accessibility of resources is generally greater in urban than rural areas [6], parks, sports and leisure might be more accessible in rural areas [15]. Considering that more than 80% of the population lives in urban areas in the most populated provinces compared to about half the population in the Atlantic provinces [16], the environments of aging Canadians differ. Also, aging Canadians living in rural areas reported a greater monthly frequency of volunteering in an organization than their urban peers [17]. In Quebec, however, two studies found no significant differences in social participation among older adults living in metropolitan, urban and rural neighborhoods but these studies involved a limited number of older participants who were not representative of the population [18, 19]. A recent 6-year longitudinal study in Germany found lower social participation in rural areas compared to urban, with more pronounced age-related decreases when there was an above-average decline in population density, suggesting fewer opportunities for social participation [20]. In addition to the rural/urban distinction, differences in social participation according to population size have been studied. In Kansas, community involvement in a civic group was positively associated with low-density rural areas [21]. A Belgian study also found less participation in communities with greater population density but only for participation in family activities and seniors’ associations [22]. To our knowledge, no Canadian study has compared the frequencies of engagement in community activities according to population size.

Social participation is also influenced by the social environment, which is defined by three broad dimensions: *interpersonal relationships* (e.g. social support and social network)*, social inequalities* (e.g. socioeconomic position and income inequality, racial discrimination), and *neighborhood and community characteristics* (e.g. social cohesion and social capital, neighborhood factors) [23]; p. 1012) In Canada, provinces could be viewed as geopolitical spaces influencing formal more than informal participation above and beyond the local community [24]. Canadian provincial governments shape the social environment in terms of support, service provision, infrastructure and policies. While the four largest Canadian provinces (Ontario, Quebec, British Columbia and Alberta) share some liberal characteristics, Alberta leans more towards an ultra-liberal or small government approach and is less inclined to invest in social and health initiatives [25]. Quebec is more akin to a European interventionist state, spending more on health care and education [25].

In addition to aspects of the physical and social environment, previous studies found gender differences in social participation; for example, older women were more likely to participate in community activities such as volunteering [26, 27] while men were more engaged in physical activities [28]. Moreover, a multiple case study with an in-depth exploration of the perspective of older adults, their families and health professionals showed that women’s and men’s social participation needs differed [19, 29]. Also, more women indicated a desire to participate in more activities [30].

To date, little is known about aging women’s and men’s social participation and barriers according to region and population size. Such a comparison is especially important for large countries like Canada that have a wide range of physical and social characteristics. Knowing how social participation and barriers to it differ according to geographical area could help to improve health and reduce the burden on public health systems, especially by fostering the creation of favorable environments. This study thus aimed to compare aging women’s and men’s social participation, desire to participate more, and barriers according to Canadian region and population size.

## Methods

### Design and participants

At the time of the study, only the cross-sectional 2008–2009 Canadian Community Health Survey - Healthy Aging (CCHS-HA) was available to address our objectives. Although the baseline of the Canadian Longitudinal Study on Aging (CLSA) was conducted in 2010–2015 [30], its data were not available at the time of the present study, and we carried out a secondary analysis of microdata from the CCHS-HA. The CCHS-HA involved face-to-face interviews with 16,274 Canadians (9730 women and 6639 men) aged 65 and over living in private dwellings (excluding people living in collective dwellings and institutions) in the ten Canadian provinces. A stratified random sampling strategy was used to recruit respondents based on age, gender, province, and rural or urban area. Information related to health, lifestyle and socioeconomic characteristics was collected. Full-time members of the Canadian Forces and residents of the three territories, Indian reserves, Crown lands and some remote regions were excluded from the sampling. A weight matrix based on age, gender, province, and rural or urban area was applied to the respondents, making the results representative of the Canadian population aged 65 and over. The use of microdata files met the stringent security and confidentiality standards set out in the *Statistics Act* to prevent individuals being identified. Data were accessed through the Quebec Interuniversity Centre for Social Statistics (QICSS) and outputs met the confidentiality standards of the *Statistics Act*. The Statistics Canada Executive Management Board, acting as the Research Ethics Board, approved the CCHS Healthy Aging component.

### Variables and measures

Sociodemographic and health characteristics considered were self-report answers to questions concerning age, annual gross household income, gender (woman/man), education [highest degree, certificate or diploma obtained: 1) less than high school graduation; 2) high school; 3) some post-secondary education; 4) post-secondary degree/diploma], driver’s license (yes/no), most common form of transportation: [1) passenger in a motor vehicle; 2) taxi; 3) public transit; 4) accessible transit; 5) cycling; 6) walking; 7) wheelchair or motorized cart; 8) driver in a motor vehicle], retirement (yes/no), living situation (people living in the household; recoded in the present study as living alone or not), immigrant (yes/no), and chronic disease (at least one chronic disease or not).

Social participation was estimated by the frequency of participation in eight community activities: *family or friends* outside the household; church or *religious*; sports or *physical*; *educational* and cultural; service *club* or fraternal organization; neighborhood, community or professional *association*; *volunteering* or charity work; and *other recreational* (e.g. hobbies and bingo). Although some of these activities can be done alone (e.g. hobbies, physical activities), the wording of the questions specified the involvement of other people (e.g. “How often did you participate in sports or physical activities that you do with other people” or “[ …] any other recreational activities involving other people”). Responses were converted into monthly frequency of engagement in each activity, i.e. “at least once a day” = 20; “at least once a week” = 6; “at least once a month” = 2; “at least once a year” = 1; and “never” = 0 [19, 31]. Frequencies were summed to give the total number of community activities per month. As measured in this study, the internal consistency of the scale was satisfactory (α = 0.72) and no item, if removed, improved it. Because the survey involved a large number of Canadians, many differences were statistically significant but should be interpreted in terms of practical differences, i.e. greater than 0.5 activities per month [19, 32].

Respondents were also asked whether they wanted to participate in more community activities (yes/no) and, if yes, give the reason(s) that prevented them from participating more, which were classified by the interviewer from a predetermined list of 13 reasons. Responses were: 1) *too busy*; 2) *health limitation*; 3) *personal or family responsibilities*; 4) *time of activities not suitable*; 5) *not wanting to go alone*; 6) *cost*; 7) *unavailability* of activities in the area; 8) *transportation* problems; 9) location *too far*; 10) afraid or concerns about *safety*; 11) *language*-related reasons; 12) location *not accessible*; and 13) *other* [not specified].

### Geographical areas

Social participation was compared by Canadian region and population size. To facilitate comparison and avoid a breach of confidentiality with small numbers of observations in several provinces, respondents were grouped based on the commonly used five-region classification: *Atlantic* (Newfoundland and Labrador, Prince Edward Island, Nova Scotia, New Brunswick); *Quebec*; *Ontario*; *Prairies* (Manitoba, Saskatchewan, Alberta); and *British Columbia*. The five-region model is also consistent with demographic projections as the proportion of seniors aged 65 and over in the grouped provinces is expected to be similar to that in the individual provinces (i.e. above the national average in the Atlantic provinces and below it in the Prairie provinces) [3]. In addition, respondents were classified by their postal code as living in one of the five population size groups: 1) *rural* (population < 1000 inhabitants or density < 400 inhabitants/km^2^); 2) *small urban* (1000–29,999 inhabitants); 3) *medium urban* (30,000-99,999); 4) *large urban* (100,000-499,999); and 5) *metropolitan* (≥500,000) areas [33].

### Analysis

Respondents were described by means or percentages, along with 95% confidence intervals. Some categories were grouped for certain variables, i.e. education: [1) less than high school graduation; 2) high school; 3) some post-secondary education or degree/diploma], and transportation: [1) driver or passenger in a motor vehicle; 2) walking or cycling; 3) public transit, accessible transit, taxi, or wheelchair or motorized cart]. Pairwise comparisons of marginal linear predictions were performed to identify statistically significant differences between categories of regions, population size groups and genders, controlling for age, income, education and transportation [34]. *P*-values were adjusted with the Bonferroni correction to limit the potential for type I errors (α = 0.005). Genders within regions or population size groups were compared against Wald tests. To account for the CCHS-HA stratified random sampling strategy, a balanced repeated replication technique was applied to compute *p*-values and 95% confidence intervals. Sampling weights were applied to enable population-level inferences. When *n*’s were too low to meet confidentiality standards for the less frequent findings, cells were removed from results tables. All statistical analyses were carried out using Stata 14.0 [35].

## Results

Respondents were between 65 and 104 years old and the women were about 1 year older on average than the men in every region (Additional file 1) and population size group except rural areas (Additional file 2). Older adults in Quebec and the Atlantic had the lowest average household incomes (Table 1) while those living in metropolises had the highest across population size groups (Additional file 2). In all Canadian regions, women had substantially lower incomes than men, i.e. between Can$9,453 and 17,317 less in the Atlantic and Prairies, respectively (Additional file 1). British Columbia had the highest proportion of respondents with at least a college education and Quebec the lowest (Table 1). Respondents living in metropolises had a greater proportion with a college education compared to rural areas (Additional file 2). By gender, men were more likely than women to have at least a college education in all regions except the Atlantic (Additional file 1), and in rural areas, large urban centers and metropolises (Additional file 2). Overall, almost three out of four respondents had a driver’s license (Table 1), with fewer women than men across all regions and population size groups (Additional files 1 and 2). Respondents in the Atlantic provinces reported a lower proportion of active transportation, i.e. walking or cycling (Table 1), and those living in metropolises relied the most on active transportation across population size groups (Additional file 2). Over nine out of ten respondents were retired in all regions but Quebec and the Atlantic had the highest proportions (Table 1). Almost half the respondents lived in metropolitan areas, followed by large urban centers and rural areas. The Atlantic had the highest share of respondents living in a rural area, and British Columbia and the Prairies the lowest. One third of respondents lived alone (Table 1), with women being more than twice as likely as men to do so (Additional file 1). Fewer rural respondents lived alone than any other population size group, except small urban centers (Additional file 2). Aging immigrants made up less than one third of respondents in Canada overall but more than two out of five in Ontario and British Columbia (Table 1) as well as metropolises (Additional file 2). Finally, more than nine in ten respondents reported having at least one chronic disease (Table 1), and women reported this more often than men in all regions and population size groups, except for the Prairies and small urban centers (Additional files 1 and Additional files 2, respectively).

**Table 1:** Socioeconomic profile by region

	Canadian region					
	B.C.	Prairies	Ontario	Quebec	Atlantic	Canada
	*n* = 2042	*n* = 3741	*n* = 3496	*n* = 2730	*n* = 4265	*n* = 16,274
Continuous variables	Mean	Mean	Mean	Mean	Mean	Mean
	[95% CI]	[95% CI]	[95% CI]	[95% CI]	[95% CI]	[95% CI]
Age (years)	74.6^B^	74.7^B^	74.7^B^	74.3^A^	74.3^A^	74.5
	[74.4,74.8]	[74.6,74.9]	[74.5,74.8]	[74.1,74.4]	[74.1,74.4]	[74.5,74.6]
Income (CAD)	49998^B^	50056^B^	54275^B^	38159^A^	40087^A^	47,822
	[45928,54067]	[47103,53010]	[50730,57819]	[35610,40707]	[37664,42511]	[46057,49587]
Categorical variables	%	%	%	%	%	%
	[95% CI]	[95% CI]	[95% CI]	[95% CI]	[95% CI]	[95% CI]
Women	53.3^B^	54.5^A^	54.9^A^	55.9^A^	55.1^A^	51.9
	[53.3,53.3]	[54.5,54.5]	[54.9,54.9]	[55.9,55.9]	[55.1,55.1]	[51.9,51.9]
Education						
Less than high school	27.7	37.4^A^	37.9^A^	54.6	47.6	41.3
	[24.9,30.6]	[35.0,39.9]	[35.4,40.5]	[51.5,57.7]	[45.0,50.2]	[39.9,42.7]
High school	18.4^B^	15.2^B^	18.0^B^	10.9^A^	11.7^a^	15.4
	[16.0,20.9]	[13.5,16.9]	[16.2,19.9]	[9.1,12.7]	[10.3,13.1]	[14.5,16.4]
College or higher	53.8	47.3^B^	44.0^AB^	34.5	40.7^a^	43.3
	[50.4,57.3]	[44.7,50.0]	[41.4,46.7]	[31.9,37.1]	[38.3,43.0]	[41.9,44.6]
Driver’s license	77.7^B^	82.0	73.3^AB^	71.3^A^	76.2^B^	74.9
	[75.3,80.2]	[80.3,83.7]	[71.0,75.5]	[69.0,73.6]	[74.4,78.0]	[73.8,76.1]
Transportation						
Car driver / passenger	83.7^B^	88.8^B^	84.2^A^	80.3	93.9^a^	84.8
	[80.7,86.8]	[87.1,90.5]	[81.7,86.7]	[77.1,83.5]	[92.6,95.1]	[83.5,86]
Walking or cycling	11.0^B^	7.0^A^	10.8^AB^	13.8^AB^	3.8	10.4
	[8.4,13.6]	[5.7,8.3]	[8.9,12.7]	[11.2,16.5]	[2.9,4.8]	[9.3,11.4]
Transit, accessible transit, taxi & wheelchair	5.2^A^	4.2^A^	5.0^A^	5.8^A^	2.3	4.9
	[3.6,6.9]	[3.2,5.2]	[3.8,6.2]	[4.3,7.3]	[1.5,3.1]	[4.3,5.5]
Retired	91.8^AB^	90.0^B^	89.6^B^	94.2^A^	94.2^A^	91.5
	[90.0,93.5]	[88.4,91.6]	[88.0,91.1]	[92.8,95.5]	[93.2,95.3]	[42.1,43.6]
Population size group						
Rural area	10.3^AB^	5.5^A^	16.8^B^	20.5^B^	29.5	16.1
	[4.2,16.3]	[3.4,7.6]	[13.5,20.1]	[16.0,25.0]	[25.7,33.4]	[14.1,18.1]
Small	6.8^AB^	5.0^AB^	2.2^A^	2.6^AB^	8.2^b^	3.8
	[2.6,11.0]	[2.7,7.3]	[0.6,3.8]	[0.5,4.6]	[5.2,11.1]	[2.7,4.9]
Medium	13.6^AB^	11.8^AB^	9.1^A^	11.0^AB^	19.3^b^	11.4
	[8.6,18.7]	[7.7,15.9]	[5.8,12.3]	[6.9,15.1]	[16.0,22.7]	[9.5,13.2]
Large	21.9^AB^	31.6^B^	25.0^B^	11.2^A^	42.9	23.4
	[15.3,28.5]	[26.6,36.6]	[19.9,30.0]	[7.1,15.3]	[38.3,47.6]	[20.9,25.9]
Metropolitan	47.4^A^	46.2^A^	47.0^A^	54.7^A^	‡	45.3
	[4.2,39.2]	[2.8,40.7]	[2.9,41.4]	[3.2,48.5]	[‡,‡]	[42.4,48.2]
Living alone	33.9^B^	35.3^AB^	36.4^AB^	39.1^A^	37.5^AB^	36.6
	[31.1,36.7]	[33.0,37.6]	[34.4,38.4]	[36.9,41.3]	[36.0,39.1]	[35.6,37.7]
Immigrant	41.5^A^	21.5	41.1^A^	12.4	5.9	28.4
	[37.9,45.0]	[19.3,23.7]	[37.6,44.5]	[9.7,15.0]	[5.0,6.9]	[26.8,29.9]
At least one chronic disease	90.8^A^	92.0^A^	91.3^A^	90.8^A^	92.9^A^	91.3
	[73.1,78.2]	[77.6,81.0]	[75.9,80.1]	[74.2,78.1]	[80.4,83.8]	[90.6,92.0]

Cells that share an uppercase letter are not significantly different (*p* > 0.05, Bonferroni adjusted); n is the unweighted sample size; ‡ no observation

Women and men participated on average in one community activity every other day, with a similar level of engagement across regions (Table 2). Overall, activities with family and friends were the most frequent for the respondents, with four or five activities per month. Mean participation with family and friends in Quebec and the Atlantic was less frequent, i.e. one less monthly activity, than the Prairies. Religious activities were the second most frequent, with British Columbia having the lowest participation (Table 2). Physical activities were the third most important community activity across the country, except for respondents in British Columbia. Prairies men also engaged more regularly in physical activities than women. Finally, respondents in the Atlantic provinces participated more often in other recreational activities than those in British Columbia (Table 2).

**Table 2:** Social participation by region and gender

Activity	Canadian region																	
	British Columbia			Prairies			Ontario			Quebec			Atlantic			Canada		
	*n* = 2042			*n* = 3741			*n* = 3496			*n* = 2730			*n* = 4265			*n* = 16,274		
	W	M	Total	W	M	Total	W	M	Total	W	M	Total	W	M	Total	W	M	Total
	%	%	%	%	%	%	%	%	%	%	%	%	%	%	%	%	%	%
	[95% CI]	[95% CI]	[95% CI]	[95% CI]	[95% CI]	[95% CI]	[95% CI]	[95% CI]	[95% CI]	[95% CI]	[95% CI]	[95% CI]	[95% CI]	[95% CI]	[95% CI]	[95% CI]	[95% CI]	[95% CI]
Family / friends	5.2^ab^	4.9^*a*^	5.0^AB^	5.4^ab^	5.4^*a*^	5.4^B^	4.9^ab^	4.8^*a*^	4.8^AB^	4.3^b^	4.4^*a*^	4.3^A^	4.6^b^	4.4^*a*^	4.5^A^	4.8	4.8	4.8
	[4.8,5.6]	[4.5,5.3]	[4.8,5.3]	[5.1,5.7]	[5.0,6.0]	[5.1,5.6]	[4.6,5.2]	[4.5,5.1]	[4.6,5.0]	[4.0,5.0]	[4.1,4.8]	[4.1,4.6]	[4.3,4.9]	[4.1,4.7]	[4.3,4.8]	[4.7,5.0]	[4.6,4.9]	[4.7,4.9]
Religious	2.1^a^	2.0^*a*^	2.1^A^	3.1^ab^	2.5^*ab*††^	2.8^BC^	2.9^bc^	2.7^*ab*^	2.8^C^	2.6^a^	2.3^*ab*^	2.5^AB^	3.5^c^	2.9^*b*†††^	3.2^C^	2.8	2.5^†††^	2.7
	[1.8,2.4]	[1.6,2.3]	[1.8,2.3]	[2.8,3.3]	[2.3,3.0]	[2.7,3.0]	[2.7,3.1]	[2.4,3.0]	[2.6,3.0]	[2.4,3.0]	[2.1,2.5]	[2.3,2.6]	[3.3,3.7]	[2.7,3.1]	[3.1,3.4]	[2.7,2.9]	[2.3,2.6]	[2.6,2.7]
Physical	2.8^b^	2.6^*abc*^	2.7^A^	2.2^a^	2.8^*a*††^	2.4^A^	2.0^ab^	2.2^*bc*^	2.1^A^	2.3^ab^	2.5^*abc*^	2.3^A^	1.8^ab^	2.0^*c*^	1.9^A^	2.2	2.4^†^	2.3
	[2.3,3.2]	[2.3,3.0]	[2.4,3.0]	[1.9,2.5]	[2.5,3.0]	[2.2,2.7]	[1.8,2.2]	[1.9,2.5]	[1.9,2.0]	[1.9,3.0]	[2.1,2.8]	[2.1,2.6]	[1.6,2.0]	[1.8,2.3]	[1.7,2.1]	[2,2.3.0]	[2.2,2.6]	[2.2,2.4]
Volunteering	1.5^a^	1.5^*ab*^	1.5^B^	1.5^ab^	1.6^*ab*^	1.5^AB^	1.3^ab^	1.2^*ab*^	1.3^AB^	1.3^a^	1.5^*a*^	1.4^B^	1.4^b^	1.5^*b*^	1.5^A^	1.4	1.4	1.4
	[1.3,1.8]	[1.2,1.8]	[1.3,1.7]	[1.4,1.7]	[1.3,2.0]	[1.4,1.7]	[1.1,1.5]	[1.0,1.4]	[1.1,1.0]	[1.1,2.0]	[1.2,1.8]	[1.2,1.6]	[1.2,1.6]	[1.3,1.8]	[1.3,1.6]	[1.3,1.5]	[1.3,1.5]	[1.3,1.5]
Educational	1.2^a^	1.1^*a*^	1.2^A^	1.1^a^	1.1^*a*^	1.1^A^	1.0^a^	0.9^*a*^	1.0^A^	1.1^a^	1.0^*a*^	1.0^B^	0.8^a^	0.8^*a*^	0.8^AB^	1.1	1.0	1.0
	[1.1,1.4]	[0.9,1.3]	[1.1,1.3]	[1.0,1.2]	[0.9,1.0]	[1.0,1.2]	[0.9,1.1]	[0.8,1.0]	[0.9,1.0]	[0.9,1.0]	[0.8,1.1]	[0.9,1.2]	[0.7,0.9]	[0.6,0.9]	[0.7,0.8]	[1.0,1.1]	[0.9,1.0]	[1.0,1.1]
Associations	1.1^a^	0.9^*a*^	1.0^A^	1.1^a^	1.0^*a*^	1.1^A^	0.8^a^	0.9^*a*^	0.9^A^	0.7^a^	0.7^*a*^	0.7^A^	0.8^a^	0.9^*a*^	0.9^A^	0.9	0.9	0.9
	[0.9,1.2]	[0.7,1.1]	[0.9,1.1]	[0.9,1.3]	[0.8,1.0]	[0.9,1.2]	[0.7,1.0]	[0.7,1.0]	[0.8,1.0]	[0.5,1.0]	[0.6,0.9]	[0.6,0.8]	[0.7,0.9]	[0.7,1.0]	[0.8,1.0]	[0.8,0.9]	[0.8,0.9]	[0.8,0.9]
Clubs	0.6^a^	0.7^*a*^	0.6^A^	0.8^a^	1.0^*a*^	0.9^A^	0.6^a^	0.7^*a*^	0.7^A^	1.1^a^	1.2^*a*^	1.1^A^	0.7^a^	1.0^*a*††^	0.8^A^	0.8	0.9	0.8
	[0.4,0.7]	[0.5,0.9]	[0.5,0.7]	[0.7,1.0]	[0.8,1.0]	[0.8,1.0]	[0.5,0.7]	[0.6,0.8]	[0.6,1.0]	[0.9,1.0]	[0.9,1.4]	[1.0,1.3]	[0.6,0.8]	[0.8,1.2]	[0.7,0.9]	[0.7,0.8]	[0.8,1.0]	[0.8,0.9]
Other recreational	1.6a	1.5^*a*^	1.5^A^	1.8^a^	1.7^*a*^	1.8^AB^	1.6^a^	1.5^*a*^	1.6^AB^	1.7^a^	1.3^*a*†^	1.5^AB^	2.1	1.8^*a*†^	1.9^B^	1.7	1.5^††^	1.6
	[1.4,1.9]	[1.2,1.7]	[1.4,1.7]	[1.7,2.0]	[1.4,2.0]	[1.6,1.9]	[1.4,1.8]	[1.3,1.7]	[1.4,2.0]	[1.5,2.0]	[1.1,1.5]	[1.4,1.7]	[1.8,2.3]	[1.6,2.0]	[1.8,2.1]	[1.6,1.8]	[1.4,1.6]	[1.5,1.7]
Total	16.0^a^	14.8^*a*^	15.5^A^	16.6^a^	16.6^*a*^	16.6^A^	15.0^a^	14.7^*a*^	14.9^A^	14.9^a^	14.8^*a*^	14.8^A^	15.5^a^	15.1^*a*^	15.3^A^	15.4	15.1	15.2
	[14.9,17.1]	[13.7,15.9]	[14.6,16.3]	[15.8,17.3]	[15.7,18]	[15.9,17.3]	[14.4,15.7]	[13.9,15.6]	[14.3,15.0]	[13.6,16.0]	[13.7,15.9]	[14.0,15.7]	[14.9,16.2]	[14.3,15.9]	[14.8,15.9]	[14.9,15.9]	[14.6,15.5]	[14.9,15.6]

Cells that share a letter are not significantly different (*p* > 0.05, Bonferroni adjusted): lowercase between W/Regions; lowercase italic between M/Regions; uppercase between Regions. † Men are significantly different than women († *p* < 0.05, †† *p* < 0.01, ††† *p* < 0.001). n is the unweighted sample size after removing missing observations

The mean participation of older adults in medium and large urban areas was greater, i.e. three and two more activities per month, respectively, than in metropolises (Table 3). Total engagement by gender did not differ across population size groups. More specifically, mean participation with family and friends of older adults living in metropolises was lower, i.e. one less monthly activity, than in medium and large urban centers (Table 3). Men in small urban centers practised about one monthly physical activity less than in medium and large urban centers. No other differences were found between population size groups and gender.

**Table 3:** Social participation by population size group and gender

Activity	Population size group																	
	Rural			Small			Medium			Large			Metropolitan			Canada		
	*n* = 2620			*n* = 619			*n* = 1855			*n* = 3808			*n* = 7372			*n* = 16,274		
	W	M	Total	W	M	Total	W	M	Total	W	M	Total	W	M	Total	W	M	Total
	%	%	%	%	%	%	%	%	%	%	%	%	%	%	%	%	%	%
	[95% CI]	[95% CI]	[95% CI]	[95% CI]	[95% CI]	[95% CI]	[95% CI]	[95% CI]	[95% CI]	[95% CI]	[95% CI]	[95% CI]	[95% CI]	[95% CI]	[95% CI]	[95% CI]	[95% CI]	[95% CI]
Family / friends	4.7^ab^	4.6^*ab*^	4.7^AB^	5.3^ab^	5.6^*ab*^	5.4^AB^	5.4^a^	5.7^*a*^	5.6^B^	5.3^a^	5.2^*a*^	5.2^B^	4.4^b^	4.3^*b*^	4.4^A^	4.8	4.8	4.8
	[4.2,5.3]	[4.3,4.9]	[4.4,5]	[4.6,6.0]	[4.5,6.6]	[4.8,6.1]	[5.1,5.8]	[5.1,6.4]	[5.2,6.0]	[5.0,5.6]	[4.8,6.0]	[5.0,5.5]	[4.2,4.6]	[4.1,4.5]	[4.2,4.5]	[4.7,5.0]	[4.6,4.9]	[4.7,4.9]
Religious	2.9^a^	2.3^*a*††^	2.6^A^	2.8^a^	2.3^*a*^	2.6^A^	3.1^a^	2.7^*a*^	2.9^A^	2.8^a^	2.4^*a*†^	2.6^A^	2.7^a^	2.6^*a*^	2.6^A^	2.8	2.5^†††^	2.7
	[2.6,3.2]	[2.0,2.6]	[2.4,2.8]	[2.4,3.0]	[1.6,2.9]	[2.2,3.0]	[2.8,3.4]	[2.3,3.1]	[2.7,3.0]	[2.6,3.0]	[2.1,3.0]	[2.5,2.8]	[2.5,2.8]	[2.4,2.8]	[2.5,2.8]	[2.7,2.9]	[2.3,2.6]	[2.6,2.7]
Physical	2.0^a^	2.4^*ab*^	2.2^A^	2.0^a^	1.7^*a*^	1.9^A^	2.4^a^	2.9^*b*^	2.6^A^	2.1^a^	2.6^*b*††^	2.3^A^	2.3^a^	2.2^*ab*^	2.3^A^	2.2	2.4^†^	2.3
	[1.6,2.4]	[1.9,2.9]	[1.8,2.5]	[1.3,3.0]	[1.2,2.1]	[1.4,2.4]	[2.0,2.8]	[2.4,3.4]	[2.3,3.0]	[1.8,2.3]	[2.3,3.0]	[2.1,2.5]	[2.0,2.5]	[2.0,2.5]	[2.1,2.4]	[2.0,2.3]	[2.2,2.6]	[2.2,2.4]
Volunteering	1.6^a^	1.4^*a*^	1.5^A^	1.4^a^	1.5^*a*^	1.4^A^	1.6^a^	1.8^*a*^	1.7^A^	1.5^a^	1.7^*a*^	1.6^A^	1.2^a^	1.2^*a*^	1.2^A^	1.4	1.4	1.4
	[1.2,1.9]	[1.1,1.7]	[1.3,1.7]	[1.0,2.0]	[1.1,1.9]	[1.2,1.7]	[1.2,2.0]	[1.3,2.3]	[1.4,2.0]	[1.3,1.6]	[1.4,2.0]	[1.4,1.7]	[1.1,1.3]	[1.0,1.3]	[1.1,1.3]	[1.3,1.5]	[1.3,1.5]	[1.3,1.5]
Educational	0.8^a^	0.8^*a*^	0.8^A^	1.3^a^	0.9^*a*^	1.1^A^	1.1^a^	1.0^*a*^	1.0^A^	1.0^a^	1.0^*a*^	1.0^A^	1.1^a^	1.0^*a*^	1.1^A^	1.1	1.0	1.0
	[0.7,1.0]	[0.7,1.0]	[0.7,0.9]	[0.8,2.0]	[0.7,1.1]	[0.9,1.4]	[0.9,1.2]	[0.9,1.1]	[0.9,1.0]	[0.9,1.1]	[0.8,1.0]	[0.9,1.0]	[1.0,1.3]	[0.9,1.1]	[1.0,1.2]	[1.0,1.1]	[0.9,1.0]	[1.0,1.1]
Associations	0.8^a^	0.9^*a*^	0.9^A^	0.8^a^	0.7^*a*^	0.8^A^	0.8^a^	0.9^*a*^	0.9^A^	1.0^a^	1.0^*a*^	1.0^A^	0.8^a^	0.8^*a*^	0.8^A^	0.9	0.9	0.9
	[0.7,1.0]	[0.7,1.1]	[0.7,1.0]	[0.6,1.0]	[0.3,1.1]	[0.5,1.0]	[0.7,1.0]	[0.6,1.2]	[0.7,1.0]	[0.8,1.1]	[0.8,1.0]	[0.9,1.1]	[0.7,1.0]	[0.7,0.9]	[0.7,0.9]	[0.8,0.9]	[0.8,0.9]	[0.8,0.9]
Clubs	1.0^a^	1.0^*a*^	1.0^A^	0.7^a^	1.0^*a*^	0.8^A^	0.8^a^	1.5^*a*†††^	1.1^A^	0.8^a^	0.9^*a*^	0.9^A^	0.7^a^	0.7^*a*^	0.7^A^	0.8	0.9	0.8
	[0.8,1.1]	[0.7,1.2]	[0.8,1.1]	[0.4,1.0]	[0.4,1.6]	[0.5,1.1]	[0.7,0.9]	[1.0,2.0]	[0.9,1.0]	[0.7,0.9]	[0.8,1.0]	[0.8,1.0]	[0.6,0.8]	[0.6,0.8]	[0.6,0.8]	[0.7,0.8]	[0.8,1.0]	[0.8,0.9]
Other recreational	1.7^a^	1.5^*a*^	1.6^A^	1.9^a^	1.3^*a*††^	1.6^A^	1.9^a^	1.8^*a*^	1.9^A^	1.8^a^	1.8^*a*^	1.8^A^	1.6^a^	1.2^*a*††^	1.4^A^	1.7	1.5^††^	1.6
	[1.4,2.0]	[1.2,1.8]	[1.4,1.8]	[1.5,2.0]	[0.9,1.6]	[1.3,1.9]	[1.7,2.2]	[1.5,2.1]	[1.7,2.0]	[1.6,2.0]	[1.6,2.0]	[1.7,2.0]	[1.4,1.7]	[1.1,1.4]	[1.3,1.5]	[1.6,1.8]	[1.4,1.6]	[1.5,1.7]
Total	15.4^a^	14.5^*ab*^	15.0^AB^	16.0^a^	14.7^*ab*^	15.4^AB^	16.9^a^	18.1^*a*^	17.4^B^	16.0^a^	16.4^*a*^	16.2^B^	14.6^a^	13.9^*b*^	14.3^A^	15.4	15.1	15.2
	[14.2,16.7]	[13.4,15.6]	[14.1,15.9]	[14.3,18.0]	[12.8,16.6]	[14.2,16.6]	[15.8,18.0]	[16.5,19.8]	[16.5,18.0]	[15.4,16.7]	[15.4,17.0]	[15.6,16.8]	[13.8,15.4]	[13.2,14.6]	[13.7,14.9]	[14.9,15.9]	[14.6,15.5]	[14.9,15.6]

Cells that share a letter are not significantly different (*p* > 0.05, Bonferroni adjusted): lowercase between W/Population size group; lowercase italic between M/Population size group; uppercase between Population size group. † Men are significantly different than women († *p* < 0.05, †† *p* < 0.01, ††† *p* < 0.001). n is the unweighted sample size after removing missing observations

About one in four respondents wanted to participate more across all regions (Table 4). Overall and in all regions, women wanted to participate more than men. For both genders, health limitations were the main barrier across regions, followed by being too busy, another personal barrier (Table 4). Men reported being too busy to participate more often than women, especially in Ontario and Quebec. Similarly, women reported not wanting to go alone more than twice as often as men, overall as well as in the Prairies, Ontario and the Atlantic (Table 4). Personal responsibilities were reported similarly across regions, except for older men in Quebec, who reported it half as often (Table 4). With respect to environmental barriers, cost, time not suitable, unavailability of activities and activities being too far away were those reported most often. No differences in environmental barriers were found by gender and region (Table 4). Barriers concerning transportation, safety, language and location not being accessible were rarely reported (data not shown).

**Table 4:** Barriers by region and gender

Barrier	Canadian region																	
	B.C.			Prairies			Ontario			Quebec			Atlantic			Canada		
	*n* = 2042			*n* = 3741			*n* = 3496			*n* = 2730			*n* = 4265			*n* = 16,274		
	W	M	Total	W	M	Total	W	M	Total	W	M	Total	W	M	Total	W	M	Total
	%	%	%	%	%	%	%	%	%	%	%	%	%	%	%	%	%	%
	[95% CI]	[95% CI]	[95% CI]	[95% CI]	[95% CI]	[95% CI]	[95% CI]	[95% CI]	[95% CI]	[95% CI]	[95% CI]	[95% CI]	[95% CI]	[95% CI]	[95% CI]	[95% CI]	[95% CI]	[95% CI]
Want to participate more	30.1^a^	22.9^*a*††^	26.7^A^	30.0^a^	24.6^*a*†^	27.5^A^	28.3^a^	21.1^*a*†††^	25.0^A^	20.4^a^	16.2^*a*†^	18.5^A^	26.9^a^	21.5^*a*††^	24.5^A^	26.6	20.7^†††^	23.9
	[26.0,34.2]	[19.2,26.6]	[23.8,29.7]	[26.9,33.2]	[21.7,27.5]	[25.5,29.6]	[25.4,31.2]	[18.1,24.0]	[22.9,27.1]	[17.6,23.2]	[13.2,19.2]	[16.4,20.6]	[24.4,29.3]	[18.8,24.2]	[22.6,26.3]	[25.0,28.2]	[19.1,22.2]	[22.8,25.1]
Personal barriers																		
Health limitation	32.0^a^	39.4^*a*^	34.9^A^	35.5^a^	27.6^*a*^	32.2^A^	37.1^a^	32.2^*a*^	35.2^A^	28.8^a^	32.5^*a*^	30.2^A^	40.9^a^	37.5^*a*^	39.5^A^	34.7	33.0	34.0
	[25.4,38.5]	[29.4,49.4]	[29.2,40.7]	[29.7,41.3]	[21.9,0.0]	[28.0,36.5]	[32.1,42.2]	[25.6,38.7]	[31.2,39.3]	[22.7,35.0]	[23.9,41.0]	[25.1,35.4]	[35.9,45.8]	[30.6,44.4]	[35.4,43.7]	[31.9,37.6]	[29.4,36.5]	[31.8,36.3]
Too busy	17.8^a^	24.6^*ab*^	20.5^A^	20.5^a^	28.0^*ab*^	23.6^A^	16.9^a^	30.7^*a*††^	22.2^A^	11.8^a^	31.9^*ab*†††^	19.4^A^	11.3^a^	16.9^*b*^	13.5^A^	16.2	28.4^†††^	20.9
	[12.1,23.5]	[14.0,35.2]	[14.8,26.3]	[14.9,26.1]	[21.1,34.9]	[19.1,28.0]	[12.5,21.4]	[24.1,37.4]	[18.6,25.8]	[6.9,16.7]	[22.3,41.4]	[14.1,24.8]	[7.4,15.3]	[11.4,22.4]	[10.3,16.7]	[13.7,18.7]	[24.6,32.2]	[18.8,23.1]
Not want to go alone	15.2^a^	15.0^*a*^	15.1^A^	22.1^a^	8.0^*a*†††^	16.3^A^	16.1^a^	7.5^*a*††^	12.9^A^	15.4^a^	8.9^*a*^	12.9^A^	15.2^a^	7.3^*a*††^	12.1^A^	16.7	9.0^†††^	13.7
	[9.0,21.3]	[7.0,23.0]	[9.8,20.4]	[16.9,27.3]	[3.2,12.7]	[12.6,20.0]	[11.5,20.8]	[4.1,10.8]	[9.8,15.9]	[10.6,20.1]	[4.2,13.7]	[9.3,16.5]	[11.5,18.9]	[4.1,10.5]	[9.5,14.7]	[14.2,19.2]	[6.8,11.3]	[12.0,15.5]
Personal responsibilities	12.1^a^	8.5^*a*^	10.7^A^	7.5^a^	9.6^*a*^	8.4^A^	10.6^a^	13.6^*a*^	11.7^A^	12.1^a^	4.6	9.2^A^	7.2^a^	10.5^*a*^	8.5^A^	10.4	10.1	10.3
	[6.8,17.5]	[3.5,13.4]	[6.7,14.6]	[4.8,10.3]	[5.5,13.6]	[6.2,10.6]	[6.7,14.5]	[7.2,20.0]	[8.4,15.0]	[4.4,19.9]	[1.3,7.9]	[4.0,14.5]	[4.3,10.1]	[3.7,17.2]	[5.3,11.6]	[8.0,12.7]	[7.2,13.0]	[8.4,12.1]
Environmental barriers																		
Cost	9.5^a^	6.4^*a*^	8.3^A^	7.4^a^	5.3^*a*^	6.6^A^	8.7^a^	10.3^*a*^	9.3^A^	9.5^a^	6.1^*a*^	8.2^A^	5.9^a^	4.4^*a*^	5.3^A^	8.6	7.5	8.2
	[4.1,15.0]	[2.3,10.5]	[4.8,11.8]	[4.3,10.6]	[2.1,8.5]	[4.3,8.8]	[4.3,13.1]	[4.4,16.3]	[5.7,12.9]	[5.6,13.4]	[2.0,10.3]	[5.4,11.0]	[3.1,8.7]	[1.6,7.2]	[3.4,7.3]	[6.4,10.7]	[4.8,10.3]	[6.5,9.9]
Time not suitable	8.1^a^	8.0^*a*^	8.1^A^	6.8^a^	8.6^*a*^	7.5^A^	8.1^a^	6.8^*a*^	7.6^A^	9.9^a^	4.2^*a*^	7.7^A^	6.7^a^	5.7^*a*^	6.3^A^	8.1	6.7	7.6
	[3.5,12.6]	[3.6,12.4]	[4.7,11.4]	[3.9,9.7]	[4.9,12.4]	[5.1,10.0]	[4.5,11.8]	[3.5,10.1]	[4.9,10.3]	[4.6,15.2]	[0.6,7.9]	[4.2,11.3]	[3.9,9.6]	[2.7,8.6]	[4.3,8.4]	[6.2,10.1]	[5.0,8.5]	[6.2,9.0]
Unavailability	5.4^a^	10.1^*a*^	7.3^A^	8.8^a^	5.4^*a*^	7.4^A^	7.5^a^	5.4^*a*^	6.7^A^	9.2^a^	8.1^*a*^	8.8^A^	11.5^a^	10.2^*a*^	11.0^A^	8.0	7.0	7.6
	[2.0,8.8]	[4.9,15.2]	[4.5,10.0]	[6.1,11.5]	[3.0,7.8]	[5.6,9.2]	[4.6,10.3]	[2.4,8.3]	[4.6,8.8]	[4.5,13.9]	[2.4,13.7]	[5.2,12.4]	[7.9,15.2]	[5.9,14.5]	[8.1,13.9]	[6.4,9.7]	[5.2,8.8]	[6.4,8.9]
Too far	6.2^a^	3.6^*a*^	5.2^A^	4.7^a^	7.1^*a*^	5.7^A^	6.3^a^	1.8^*a*†^	4.6^A^	5.1^a^	6.6^*a*^	5.7^A^	4.0^a^	5.1^*a*^	4.4^A^	5.6	4.2	5.0
	[2.0,10.5]	[0.0,7.4]	[2.4,8.0]	[2.4,7.1]	[1.6,12.6]	[3.1,8.3]	[3.0,9.5]	[0.1,3.4]	[2.5,6.6]	[1.8,8.4]	[1.6,11.5]	[2.9,8.4]	[2.2,5.7]	[2.2,8.0]	[2.9,5.9]	[4.0,7.2]	[2.5,5.9]	[3.8,6.3]
Other	10.4^a^	13.8^*a*^	11.7^A^	9.7^a^	17.5^*a*†^	12.9^A^	9.0^a^	12.8^*a*^	10.5^A^	7.2^a^	8.9^*a*^	7.8^A^	7.9^a^	10.8^*a*^	9.1^A^	8.9	12.9^††^	10.4
	[5.0,15.8]	[7.0,20.5]	[7.3,16.2]	[6.7,12.8]	[12.2,22.7]	[10.0,15.8]	[6.0,12.0]	[8.6,16.9]	[8.0,12.9]	[3.5,10.9]	[3.9,13.9]	[4.7,10.9]	[5.4,10.5]	[6.4,15.3]	[6.7,11.4]	[7.2,10.6]	[10.5,15.3]	[9.1,11.8]

Cells that share a letter are not significantly different (*p* > 0.05, Bonferroni adjusted): lowercase between W/Region; lowercase italic between M/Region; uppercase between Region. † Men are significantly different than women († *p* < 0.05, †† *p* < 0.01, ††† *p* < 0.001). n is the unweighted sample size after removing missing observations

Proportions of respondents wanting to participate more did not differ across population sizes (Table 5). Overall, more women than men wanted to increase their participation, especially in metropolises and medium urban centers. Personal barriers did not differ between population size groups. Men were much more likely than women to report being too busy, except in medium urban centers (Table 5). Conversely, men reported substantially less than women not wanting to go alone in medium urban centers and metropolises. Concerning environmental barriers, the unavailability of activities was reported by more than one in ten respondents in rural areas, which was twice the proportion found in metropolises (Table 5). Rural women also reported unavailability much more often than women living in metropolises, medium and large urban centers. Transportation problems were at least ten times more frequent for women than men in rural areas. Other barriers were reported similarly across genders and population size groups (Table 5). However, men in large urban centers and metropolises reported transportation problems more often than rural men. Barriers concerning cost, location being too far, concerns about safety, language and location not being accessible were rarely reported (data not shown).

**Table 5:** Barriers by population size group and gender

Barrier	Population size group																	
	Rural			Small			Medium			Large			*Metropolitan*			Canada		
	*n* = 2620			*n* = 619			*n* = 1855			*n* = 3808			*n* = 7372			*n* = 16,274		
	W	M	Total	W	M	Total	W	M	Total	W	M	Total	W	M	Total	W	M	Total
	%	%	%	%	%	%	%	%	%	%	%	%	%	%	%	%	%	%
	[95% CI]	[95% CI]	[95% CI]	[95% CI]	[95% CI]	[95% CI]	[95% CI]	[95% CI]	[95% CI]	[95% CI]	[95% CI]	[95% CI]	[95% CI]	[95% CI]	[95% CI]	[95% CI]	[95% CI]	[95% CI]
Want to participate more	23.1^a^	19.9^*a*^	21.6^A^	25.0^a^	21.9^*a*^	23.7^A^	25.1^a^	18.3^*a*†^	22.1^A^	28.0^a^	22.4^*a*†^	25.6^A^	27.6^a^	20.6^*a*†††^	24.4^A^	26.6	20.7^†††^	23.9
	[19.3,27.0]	[16.1,23.7]	[18.8,24.4]	[17.6,32.5]	[14.4,29.4]	[19.1,28.2]	[21.2,28.9]	[14.7,22.0]	[19.5,24.7]	[25.1,30.9]	[19.2,25.6]	[23.5,27.6]	[25.1,30.1]	[18.3,22.9]	[22.6,26.2]	[25.0,28.2]	[19.1,22.2]	[22.8,25.1]
Personal barriers																		
Health limitation	39.5^a^	29.3^*a*^	35.0^A^	42.3^a^	23.7^*a*†^	34.7^A^	40.7^a^	37.1^*a*^	39.4^A^	36.3^a^	32.9^*a*^	35.0^A^	30.5^a^	34.3^*a*^	31.9^A^	34.7	33.0	34.0
	[29.5,49.6]	[19.1,39.5]	[27.5,42.5]	[31.4,53.2]	[12.5,34.8]	[25.7,43.8]	[32.4,49.0]	[27.1,47.0]	[32.9,45.8]	[31.1,41.5]	[26.3,39.5]	[31.0,39.0]	[26.4,34.5]	[28.9,39.7]	[28.6,35.3]	[31.9,37.6]	[29.4,36.5]	[31.8,36.3]
Too busy	6.5^a^	27.1^*a*†††^	15.6^A^	8.1^a^	24.6^*a*†^	14.8^A^	14.3^a^	24.0^*a*^	17.8^A^	15.9^a^	29.5^*a*††^	21.1^A^	20.2^a^	29.6^*a*††^	23.8^A^	16.2	28.4^†††^	20.9
	[1.6,11.5]	[16.7,37.5]	[9.4,21.8]	[0.6,15.6]	[9.8,39.5]	[6.6,23.1]	[8.0,20.6]	[15.1,32.8]	[12.6,23.0]	[11.0,20.9]	[21.9,37.0]	[16.8,25.3]	[16.3,24.2]	[24.1,35.2]	[20.6,27.1]	[13.7,18.7]	[24.6,32.2]	[18.8,23.1]
Do not want to go alone	17.4^a^	6.4^*a*^	12.5^A^	11.0^a^	6.3^*a*^	9.1^A^	15.4^a^	6.3^*a*†^	12.1^A^	15.6^a^	11.0^*a*^	13.8^A^	18.0^a^	9.7^*a*††^	14.8^A^	16.7	9.0^†††^	13.7
	[7.4,27.4]	[1.9,10.9]	[6.6,18.4]	[3.2,18.7]	[0.0,14]	[3.8,14.4]	[8.5,22.4]	[1.9,10.8]	[7.5,16.7]	[12.2,18.9]	[6.2,15.9]	[11.0,16.7]	[14.1,21.8]	[6.2,13.2]	[12.1,17.6]	[14.2,19.2]	[6.8,11.3]	[12.0,15.5]
Personal responsibilities	5.7^a^	16.6^*a*^	10.5^A^	15.7^a^	16.2^*a*^	15.9^A^	7.8^a^	7.2^*a*^	7.5^A^	11.8^a^	6.8^*a*^	9.9^A^	11.1^a^	9.6^*a*^	10.5^A^	10.4	10.1	10.3
	[2.6,8.9]	[4.6,28.5]	[4.7,16.4]	[2.9,28.6]	[2.5,29.9]	[6.4,25.5]	[3.5,12.1]	[2.3,12.1]	[4.4,10.7]	[7.6,16.0]	[3.4,10.2]	[7.0,12.8]	[6.8,15.3]	[6.1,13.2]	[7.5,13.6]	[8.0,12.7]	[7.2,13.0]	[8.4,12.1]
Environmental barriers																		
Time not suitable	4.4^a^	8.8^*a*^	6.3^A^	3.9^a^	10.7^*a*^	6.7^A^	5.8^a^	1.4^*a*†^	4.2^A^	5.4^a^	6.5^*a*^	5.8^A^	11.6^a^	6.9^*a*†^	9.8^A^	8.1	6.7	7.6
	[1.5,7.3]	[3.0,14.6]	[3.2,9.5]	[0.1,7.6]	[0.0,22.1]	[1.0,12.3]	[1.8,9.8]	[0.0,2.9]	[1.5,6.9]	[2.4,8.5]	[3.6,9.3]	[3.6,8.0]	[8.0,15.3]	[4.2,9.7]	[7.3,12.4]	[6.2,10.1]	[5.0,8.5]	[6.2,9.0]
Unavailability	15.5^a^	11.2^*a*^	13.6^B^	3.9^ab^	9.6^*ab*^	6.2^AB^	5.3^b^	10.2^*ab*^	7.1^AB^	7.4^b^	7.7^*ab*^	7.5^AB^	7.2^b^	4.1^*b*^	6.0^A^	8.0	7.0	7.6
	[9.1,22.0]	[5.2,17.3]	[9.2,18.0]	[0.0,9.0]	[0.2,19.0]	[1.5,10.9]	[2.7,7.8]	[1.7,18.7]	[3.2,10.9]	[4.7,10.1]	[4.1,11.3]	[5.6,9.5]	[4.7,9.7]	[2.0,6.2]	[4.3,7.8]	[6.4,9.7]	[5.2,8.8]	[6.4,8.9]
Transportation	15.1^a^	1.2^*a*†††^	8.9^A^	8.0^ab^	‡	4.7^A^	13.3^ab^	1.4^*ab*†††^	9.0^A^	10.8^ab^	2.7^*b*†††^	7.7^A^	10.1^b^	6.0^*b*†^	8.5^A^	‡	‡	‡
	[8.6,21.6]	[0.0,2.4]	[5.3,12.6]	[1.6,14.4]	[‡,‡]	[1.0,8.5]	[8.5,18.1]	[0.0,3.2]	[5.6,12.3]	[7.4,14.2]	[1.1,4.2]	[5.5,9.9]	[7.2,12.9]	[3.0,9.0]	[6.3,10.7]	[‡,‡]	[‡,‡]	[‡,‡]
Other	4.8^a^	5.8^*a*^	5.3^A^	16.4^a^	21.7^*a*^	18.5^A^	13.2^a^	16.1^*a*^	14.2^A^	8.8^a^	12.6^*a*^	10.3^A^	8.5^a^	14.2^*a*^	10.6^A^	8.9	12.9^††^	10.4
	[1.5,8.1]	[2.2,9.5]	[2.8,7.8]	[6.3,26.5]	[1.6,41.7]	[8.1,29.0]	[7.1,19.3]	[9.1,23.1]	[9.8,18.7]	[6.1,11.6]	[7.8,17.5]	[7.8,12.7]	[5.9,11.1]	[10.6,17.7]	[8.5,12.8]	[7.2,10.6]	[10.5,15.3]	[9.1,11.8]

Cells that share a letter are not significantly different (*p* > 0.05, Bonferroni adjused): lowercase between W/Population size group; lowercase italic between M/Population size group; uppercase between Population size group. † Men are significantly different than women († *p* < 0.05, †† *p* < 0.01, ††† *p* < 0.001). n is the unweighted sample size after removing missing observations. ‡ values could not be released

## Discussion

This study compared social participation, desire to participate more, and perceived barriers to participation of aging women and men according to Canadian region and population size. Results showed that Canadians engage in about one community activity every other day, with very few large differences between regions, population sizes and genders. The desire to participate more, indicating the presence of at least one barrier to social participation, was expressed by one quarter of respondents. Perceived personal and environmental barriers were mostly uniform across regions, population sizes and genders.

### Social participation, motivation and desire to participate more

In this study, very few differences in social engagement were observed among Canadian provinces. Previous studies in Quebec did not find any significant differences between living environments [18, 19] but they used different measures of social participation and a less comprehensive typology of living environments. Based on the population size comparisons in the present study, the environment of local communities could nevertheless be a greater determinant of social participation than the province. A recent study in Wisconsin found that respondents in rural counties were 30% more likely to report low levels of social participation than those in urban counties (*p* < 0.05) [36]. Conversely, an Iowa study showed that rural older adults had more social interactions (on a scale from 4 to 12, mean = 7.37; SD = 2.55) than urban older adults (mean = 6.40; SD = 2.53; *p* < 0.05) [37]. However, in the Wisconsin study, the rural-urban classification was based on population density rather than population size, and the respondents were a very specific population (mostly white men and women who graduated from Wisconsin high schools in 1957). In addition, the Iowa study used a convenience sample, and four questions answered on a three-point scale would not have been enough to assess the complexity of social participation.

Previous analyses of the same survey showed that fewer older Canadians than those aged 45–64 wanted to participate more (23.9% [95%CI = 22.8;25.1] vs 36.8% [95%CI = 33.2;37.4]; *p* < 0.001) [28], plausibly because older adults optimize their resources by selecting the most salient activities in their lives [38]. By adapting their behaviors to counteract their barriers, older adults may not have the motivation to engage in additional social activities, which may explain the lower percentage of older adults who want to participate more. In addition, as they get closer to the end of life, older adults might experience a shift in motivation, decreasing their involvement in social activities and prioritizing meaningful social relationships, such as with family members, rather than more superficial social activities [39]. Nonetheless, one fourth of Canadian respondents reported wanting to participate more, which suggests that the average frequency of participation could be higher, especially if personal and environmental barriers are removed.

### Need for local strategies

Provincial institutions and the social environment did not seem to increase inequalities since older adults reported personal and environmental barriers similarly across all regions. Locally, however, some differences were found among respondents in different population size groups. Similarly to previous studies indicating that greater accessibility to resources and services increased social participation in Quebec’s urban and metropolitan areas [18, 19], the present study found that unavailability of activities was reported twice as often overall in rural than metropolitan areas. This difference in the availability of resources reinforces the need for strategies at the local community level. For example, multiple uses of the same site (e.g. library, post office, art classes) and community development initiatives (e.g. skills development, caregiving) tailored to the specific population are recommended [40]. The recent World Health Organization’s (WHO) promotion of age-friendly communities may have improved access to resources and transportation [41], which would not be shown in these data collected in 2008–2009. Also, the 2012–2017 Quebec Action Plan entitled *Aging and Living Together: At Home, In One’s Community*, promoted social participation and transportation initiatives in Quebec [42].

### Gender differences

Strategies fostering social participation should target gender differences [45], especially while considering the local community, transportation, and diversity of activities. Based on the results of this study and with respect to their local environment, women and men perceived barriers differently. Not wanting to go alone to an activity was reported more often by women than men, especially in metropolitan areas, and underlying explanations, such as fear of rejection and exploitation [46], should be investigated in future studies. In the meantime, efforts could be made to foster social participation. For example, with the personalized citizen assistance for social participation, which has been shown to improve participation in leisure activities (frequency of activities went from 9 (Q = 3.5) to 14 (Q = 3.1) out of 30; *p* < 0.01), older adults are paired with community attendants helping them to do activities in the community [47].

Among other barriers in this study, older women across the country reported transportation problems, especially in rural areas. Previous results from the same survey showed less social participation by older women without a driver’s license [28]. According to another study comparing households with and without a licensed driver, older Californians living in a household without a licensed driver were 1.6 times more likely to report severely reduced mobility for social and recreational destinations (*p* < 0.001) [43]. Given the importance of driving, the availability of public transit for older adults living in rural areas is an issue. Indeed, results from the present survey found that, outside metropolitan areas, half of older Canadians who needed help to get to places outside walking distance mentioned not using accessible transit because this service was not available [44]. For an issue like transportation, collaboration between governments should be sought, not only for healthcare destinations but also for recreational opportunities [45].

Lastly, strategies encouraging social participation should promote and include a wide variety of activities, including their positive influence on health, especially for men. In a previous study, men were found to be unaware of and did not actively look for social opportunities [46]. In the present study, men were generally more likely than women to report being too busy, particularly in smaller communities. Gendered activities, such as Men’s Sheds [51], have been shown to improve men’s mental health and social well-being, and could be promoted as a positive strategy to increase older men’s social engagement.

### Strengths and limitations

This secondary analysis of a large Canadian survey broadened our understanding of aging Canadians’ social participation and identified the main perceived barriers to engagement in community activities according to Canadian region and population size. The sampling strategy aimed to achieve demographic and geographical representativeness and the bootstrap resampling technique favored generalization and improved comparisons. With increased awareness of regional and local challenges related to community activities, decision-makers will be better equipped to plan strategies to reduce restrictions on older Canadians’ social participation.

This study has some limitations. The CCHS-HA surveyed Canadians in 2008–2009, which might not reflect the social participation of current generations. Indeed, a Dutch longitudinal study found increases in both formal and informal participation between two aging cohorts 10 years apart, mostly explained by education level [46]. In Canada in 2015, younger aging cohorts had higher education levels than older cohorts [47]. In addition, the median retirement age of Canadians rose between 2008 and 2018 [48], which may have increased the proportion of older adults who reported being too busy as a barrier for participation. Retirement at a later age also provides more income and possibly greater access to a car, both of which are associated with higher social participation levels [49]. The CCHS-HA is a cross-sectional survey and, as with other correlational studies, this precludes any appraisal of the directionality of associations. A longitudinal study could provide insight into changes in the frequency of social participation and barriers after a demographic changes or move to a different environment [20]. Finally, aggregating respondents within a five-region classification rather than by province meant that some provincial differences could not be taken into consideration, such as inequities in healthcare [50]. To avoid breaching confidentiality when there were small numbers of observations, the provinces were still aggregated even if small but significant differences in barriers were observed between the aggregated provinces, especially in the Prairies (data not shown).

## Conclusion

In Canada, population aging calls for innovative and sustainable solutions, including the creation of environments conducive to improving older adults’ health and quality of life and enabling them to have a full and rewarding social life. This study sheds light on aging Canadians’ participation in community activities and perceived barriers, according to region, population size and gender. Differences between regions and population sizes in the frequency of monthly participation in community activities were small. However, barriers in the environment suggest that local strategies targeting the social participation of older women and men might be more effective than generalized approaches. Transportation challenges, for example, could be prioritized in smaller communities than metropolises. Our findings could provide knowledge and insight for future research, such as an in-depth analysis of factors restricting social participation. For further informative comparisons about the local environment, subsequent analyses should include indicators of environmental characteristics (neighborhood social cohesion [51], availability of specific resources and services such as public transit, parks, social and material deprivation, etc.), and control for confounding factors and interactions.

## Additional files

Additional file 1: Gender distribution by region. (XLSX 17 kb)

Additional file 2: Gender distribution by population size group. (XLSX 16 kb)

## Data Availability

The data that support the findings of this study are available from Statistics Canada but some restrictions apply; these data were used under license for the present study and are not publicly available. Application process and guidelines are available from the Research Data Centres (RDC) Program (http://www.statcan.gc.ca/eng/rdc/process).

## Abbreviations

95%CI: 95% confidence interval
BC: British Columbia
CCHS-HA: Canadian Community Health Survey - Healthy Aging
CLSA: Canadian Longitudinal Study on Aging
Q: Semi-Interquartile Range
QICSS: Quebec Interuniversity Centre for Social Statistics
SD: Standard deviation

## References

[1] United Nations (2000). Replacement migration: is it a solution to declining and ageing populations?.

[2] Statistics Canada (2014). Table 051–0001 - estimates of population, by age group and sex for July 1, Canada, provinces and territories, annual.

[3] Statistics Canada (2014). Population projections: Canada, the provinces and territories, 2013 to 2063.

[4] LaPierre TA, Hughes ME, Uhlenberg P (2009). Population aging in Canada and the United States. International handbook of population aging.

[5] Levasseur M, Richard L, Gauvin L, Raymond E (2010). Inventory and analysis of definitions of social participation found in the aging literature: proposed taxonomy of social activities. Soc Sci Med.

[6] Bowling A, Stafford M (2007). How do objective and subjective assessments of neighbourhood influence social and physical functioning in older age? Findings from a British survey of ageing. Soc Sci Med.

[7] Holt-Lunstad J, Smith TB, Layton JB. Social relationships and mortality risk: a meta-analytic review. PLoS Med. 2010;7(7):1–20. 10.1371/journal.pmed.1000316 PMC2910600 20668659

[8] Wilkins K. Social support and mortality in seniors. Health Rep. 2003;14(3):23–37. 12816013

[9] Lund R, Nilsson CJ, Avlund K (2010). Can the higher risk of disability onset among older people who live alone be alleviated by strong social relations? A longitudinal study of non-disabled men and women. Age Ageing.

[10] Glass TA, De Leon CFM, Bassuk SS, Berkman LF (2006). Social engagement and depressive symptoms in late life longitudinal findings. J Aging Health.

[11] Glei DA, Landau DA, Goldman N, Chuang Y-L, Rodriguez G, Weinstein M (2005). Participating in social activities helps preserve cognitive function: an analysis of a longitudinal, population-based study of the elderly. Int J Epidemiol.

[12] Newall NE, McArthur J, Menec VH (2014). A longitudinal examination of social participation, loneliness, and use of physician and hospital services. J Aging Health.

[13] Davison KK, Lawson CT (2006). Do attributes in the physical environment influence children's physical activity? A review of the literature. Int J Behav Nutr Phys Act.

[14] Levasseur M, Généreux M, Bruneau J-F, Vanasse A, Chabot É, Beaulac C, Bédard M-M (2015). Importance of proximity to resources and to recreational facilities, social support, transportation and neighborhood security for mobility and social participation in older adults: results from a scoping study. BMC Public Health.

[15] Pearce J, Witten K, Bartie P (2006). Neighbourhoods and health: a GIS approach to measuring community resource accessibility. J Epidemiol Community Health.

[16] Statistics Canada (2011). 2011 census of population.

[17] Turcotte M (2005). Social engagement and civic participation: are rural and small town populations really at an advantage?.

[18] Therrien FH, Desrosiers J (2010). Participation of metropolitan, urban and rural community-dwelling older adults. Arch Gerontol Geriatr.

[19] Levasseur M, Cohen AA, Dubois M-F, Généreux M, Richard L, Therrien F-H, Payette H (2015). Environmental factors associated with social participation of older adults living in metropolitan, urban, and rural areas: the NuAge study. Am J Public Health.

[20] Huxhold O, Fiori KL. Do demographic changes jeopardize social integration among aging adults living in rural regions? J Gerontol Series B. 2018:1-10. 10.1093/geronb/gby008 PMC6703232 29420796

[21] Greiner KA, Li C, Kawachi I, Hunt DC, Ahluwalia JS (2004). The relationships of social participation and community ratings to health and health behaviors in areas with high and low population density. Soc Sci Med.

[22] Hooghe Marc, Botterman Sarah (2011). Urbanization, Community Size, and Population Density. Nonprofit and Voluntary Sector Quarterly.

[23] McNeill LH, Kreuter MW, Subramanian S (2006). Social environment and physical activity: a review of concepts and evidence. Soc Sci Med.

[24] Gaudet S, Turcotte M (2013). Sommes-nous égaux devant l’«injonction» à participer? Analyse des ressources et des opportunités au cours de la vie. Sociol Soc.

[25] Bernard P, Saint-Arnaud S (2004). Du pareil au même? La position des quatre principales provinces canadiennes dans l'univers des régimes providentiels. Can J Sociol.

[26] Turcotte M (2006). Seniors’ access to transportation. Can Soc Trends.

[27] Gilmour H (2012). Social participation and the health and well-being of Canadian seniors. Health Rep.

[28] Naud D, Levasseur M, Rowe KT (2015). Social participation and environmental barriers among aging Canadians: distribution and differences in gender, age and location. Social isolation, participation and impact on mental health.

[29] Turcotte P-L, Côté C, Coulombe K, Richard M, Larivière N, Couture M (2015). Social participation during transition to adult life among young adults with high-functioning autism Spectrum disorders: experiences from an exploratory multiple case study. Occup Ther Ment Health.

[30] Kirkland SA, Griffith LE, Menec V, Wister A, Payette H, Wolfson C, Raina PS (2015). Mining a unique Canadian resource: the Canadian longitudinal study on aging. Can J Aging.

[31] Richard L, Gauvin L, Gosselin C, Laforest S (2009). Staying connected: neighbourhood correlates of social participation among older adults living in an urban environment in Montreal**,** Quebec. Health Promot Int.

[32] Levasseur M, Gauvin L, Richard L, Kestens Y, Daniel M, Payette H (2011). Associations between perceived proximity to neighborhood resources, disability, and social participation among community-dwelling older adults: results from the VoisiNuAge study. Arch Phys Med Rehabil.

[33] Statistics Canada (2015). Population Centre (POPCTR): detailed definition.

[34] Tabachnick BG, Fidell LS. Using multivariate statistics. Boston: Allyn & Bacon/Pearson Education; 2007.

[35] StataCorp (2017). Stata Statistical Software: Release 15.

[36] Vogelsang EM (2016). Older adult social participation and its relationship with health: rural-urban differences. Health Place.

[37] Evans RJ (2009). A comparison of rural and urban older adults in Iowa on specific markers of successful aging. J Gerontol Soc Work.

[38] Lang FR, Rieckmann N, Baltes MM (2002). Adapting to aging losses: do resources facilitate strategies of selection, compensation, and optimization in everyday functioning?. J Gerontol Ser B Psychol Sci Soc Sci.

[39] Carstensen LL, Charles ST (1998). Emotion in the second half of life. Curr Dir Psychol Sci.

[40] Clark KJ, Leipert BD (2012). Strengthening and sustaining social supports for rural elders. Online J Rural Nurs Health Care.

[41] Orpana H, Chawla M, Gallagher E, Escaravage E (2016). Developing indicators for evaluation of age-friendly communities in Canada: process and results. Health Promot Chronic Dis Prev Can.

[42] Moulaert T. Quebec’s Vieillir et vivre ensemble Policy on Ageing: A critical outside analysis. Can Rev Soc Policy. 2012;68/69:125.

[43] Taylor BD, Tripodes S (2001). The effects of driving cessation on the elderly with dementia and their caregivers. Accid Anal Prev.

[44] Turcotte M (2012). Profile of seniors’ transportation habits. Can Soc Trends.

[45] Ryser L, Halseth G (2012). Resolving mobility constraints impeding rural seniors' access to regionalized services. J Aging Soc Policy.

[46] Van Groenou MB, Deeg DJ (2010). Formal and informal social participation of the ‘young-old’in the Netherlands in 1992 and 2002. Ageing Soc.

[47] Statistics Canada (2017). Working seniors in Canada: census in brief.

[48] Statistics Canada (2018). Retirement age by class of worker, annual.

[49] Bowling A. Ageing well: Quality of life in old age. Maidenhead: McGraw-Hill Education; 2005.

[50] Allin Sara (2008). Does Equity in Healthcare Use Vary across Canadian Provinces?. Healthcare Policy | Politiques de Santé.

[51] Windsor TD, Fiori KL, Crisp DA (2011). Personal and neighborhood resources, future time perspective, and social relations in middle and older adulthood. J Gerontol Ser B.

